# The Role of Macrolides in Childhood Non-Cystic Fibrosis-Related Bronchiectasis

**DOI:** 10.1155/2012/134605

**Published:** 2012-04-18

**Authors:** R. Masekela, R. J. Green

**Affiliations:** Paediatric Pulmonology Division, Department of Paediatrics and Child Health, Steve Biko Academic Hospital, University of Pretoria, Pretoria 0001, South Africa

## Abstract

Non-cystic fibrosis-related bronchiectasis is a chronic inflammatory lung disease, which is regarded as an “orphan” lung disease, with little research devoted to the study of this condition. Bronchiectasis results in impaired quality of life and mortality if left untreated. The tools available in the armamentarium for the management of bronchiectasis entail antibiotic therapy traditionally used to treat exacerbations, stratagems to improve mucociliary clearance, and avoidance of toxins. Macrolides have been known for the last two decades to have not only anti-bacterial effects but immunomodulatory properties as well. In cystic fibrosis, the use of macrolides is well documented in subjects colonized with *Pseudomonas aeruginosa*, to improve quality of life and lung function. There is currently emerging evidence to suggest the benefit of macrolides in subjects not colonized with *Pseudomonas aeruginosa*. This beneficial effect has been less explored in the context of bronchiectasis from other causes. The purpose of this paper is to review the current literature on the use of macrolides in non-cystic fibrosis related bronchiectasis in paediatrics.

## 1. Bronchiectasis

The term bronchiectasis is derived from the Greek words *bronkia* (bronchial tubes), *ek* (out), and *tasis* (stretching). The earliest description of bronchiectasis was by Laennac in 1819 [1]. There are two anatomical classification systems used for the diagnosis of bronchiectasis, namely, the Reid and Whitwell classifications [2, 3]. In the past few years, the diagnostic criteria for bronchiectasis have changed, with the diagnosis being based on the less invasive high-resolution computerized tomography (HRCT). HRCT scanning has led to easier diagnosis and follow up of bronchiectasis [4].

**Figure 1:**
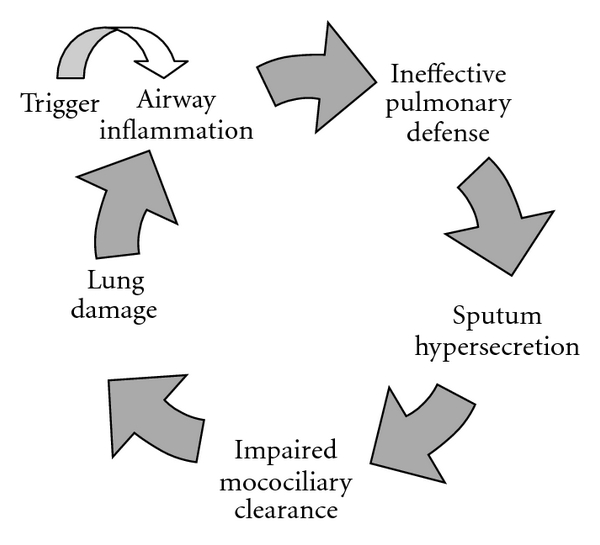
The pathophysiology of bronchiectasis the inflammatory cycle as proposed by Cole.

The exact pathophysiological mechanisms for bronchiectasis are unknown, with the currently accepted concept being the “vicious cycle” theory proposed by Cole in the mid-eighties (Figure 1) [5]. Cole's theory evolves around an initial “hit” or trigger that results in airway inflammation. The inflammatory process is established such that, with subsequent lung infections, persistent airway inflammation occurs. This is associated with release of proinflammatory cytokines interleukin-(IL-) 6, IL-8, and neutrophil elastases [6–8]. These cytokines recruit inflammatory mediators, whose end-product is mucous gland hypertrophy and mucus hyperproduction. Excess mucus compromises the mucociliary escalator, which further perpetuates microbial invasion of the airway. Mucus performs an innate immune function property in the lungs by acting as the first barrier in the airways. Mucus is made up of mucin proteins, water, surfactant phospholipids, peptides, and defence proteins. There are many changes that occur to the mucus properties of patients with chronic inflammatory lung disease [9]. There is goblet cell hyperplasia, which contributes to excessive mucus production. In the presence of infection epithelial cells modulate the recruitment of inflammatory cells by the production of chemokines, cytokines, adhesion molecules, and modulation of expression of receptors. The presence of persistent infection, impairment of the protective mucociliary escalator, and the presence of enzymes such as elastases cause damage to the airway and lung tissue [10].

Risk factors associated with bronchiectasis are overcrowding, poverty, damp housing, macro- and micro-malnutrition, indoor pollution with biomass fuels, and environmental tobacco smoke. These risks factors have been largely diminished in developing countries with rates of bronchiectasis as low as 0.49 per 100 000 population in Finnish children [11–13]. Certain groups in developed countries, such as the Alaskan natives of the Yokun Kuskokwim Delta, the New Zealand Maori, and the Aborigines of Australia, have inordinately high bronchiectasis rates, ranging from 3.5 to 16 per 10 000 [14–16]. This is in contradistinction to developing countries where there is a high infectious disease burden and consequently high bronchiectasis rates [17]. There is, however, no accurate prevalence data available to quantify the problem in developing countries.

## 2. Immunology of Bronchiectasis

The innate immune system is activated by pathogen-associated molecular patterns (PAMPs), which are recognized by pattern recognition receptors such as toll-like receptors (TLRs) [18, 19]. TLR activation triggers a cascade resulting in the activation and nuclear translocation of nuclear factor *κβ* (NF*κβ*) with subsequent release of proinflammatory cytokines IL-1*β*, IL-8, and TNF-*α* [20]. IL-8 is a potent chemoattractant for neutrophils [21]. Neutrophils are integral to the innate immune mechanisms in the lung, with neutrophillic inflammation being central in the pathogenesis of bronchiectasis. Elevated levels of neutrophil derived products IL-6, IL-8 and TNF-*α* have been found in the sputum of adults with stable bronchiectasis [22]. Transepithelial migration of neutrophils from the intravascular compartment occurs in a coordinated fashion with interplay of various adhesion molecules. Three families of adhesion molecules mediate this; the selectins, the integrins CD11/CD18, and the immunoglobulin superfamily that is, intravascular adhesion molecule 1 (ICAM-1) and vascular adhesion molecule 1 (VCAM-1) [23]. These adhesion molecules are upregulated in the presence of IL-1, TNF-*α*, and IL-8. Both VCAM-1 and ICAM-1 have been found to be elevated in bronchiectasis subjects [10]. Adherent neutrophils migrate to the inflammatory site under the direction of the neutrophil chemoattractant IL-8. Once activated, neutrophils produce neutrophil elastase (NE) and matrix metalloproteinases: MMP-8 and MMP-9. NE has three main mechanisms of action. Firstly, it has proteolytic effects from toxic products that digest the airway elastin, basement membrane collagen, and proteoglycan [23]. Secondly, it induces the release of cytokines IL-6, IL-8, and GM-CSF [23]. Finally, it is a powerful secretagogue inducing expression of mucin gene MUC5AC via the generation of reactive oxygen species [23]. In CF, the free elastase is associated with reduced opsonization of pathogens, thus acting as a potent stimulator for IL-8 production [24].

Granulocyte-macrophage colony-stimulating factor (GM-CSF) is a potent chemokine that allows prolonged survival of neutrophils in the airway. The intensity of the proinflammatory cytokines was also found to be elevated in subjects with colonization of the airways by microorganisms. This elevation in the cytokines, coupled with the elevated proteases released from neutrophils, namely, neutrophil elastase, MMP-2, MMP-6, and MMP-9, overwhelms the antiprotease defence mechanisms rendering the lung vulnerable to destruction [25–27]. The use of antibiotics has been shown to result in a reduction of these proinflammatory cytokines [28].

## 3. Management of Bronchiectasis

Interventions in the management of bronchiectasis include medical as well as adjunctive therapies. The therapeutic goals of management include the following: treatment of the underlying disease, aggressive treatment of infections, promotion of mucociliary clearance, promotion of normal growth, avoidance of toxins, identification and management of complications, and treatment of the chronic inflammation to retard disease progression [29].

Although airway clearance with chest physiotherapy is universally recommended the evidence for benefit is limited. A Cochrane review demonstrated no improvement in lung function in patients who had regular multimodality airway clearance techniques [30]. The benefit to individuals seems to lie in the reduction of cough frequency and improvement in quality of life. The technique used does not appear to have any impact on the outcome, although in patients with gastroesophageal reflux, care should be taken when instituting techniques that use the head down position. This is particularly important in young children. There have been no favourable outcomes, in terms of lung function parameters, with the use of physiotherapy [31].

In bronchiectasis, the rheological properties of mucus are abnormal with variation in the rheology depending on the cause of bronchiectasis. In childhood, postinfective bronchiectasis mucus is less viscous and more transportable than that of children with CF [32]. The agents used for airway clearance are either airway hydrators or mucolytics. Mucolytic agents reduce mucus viscosity and promote clearance of secretions. They do this via several mechanisms, which include disruption of disulphide bonds and liquefying proteins that degrade DNA filaments and actin. This modality of treatment is an attractive option in a condition where increased mucus tenacity and viscosity is a problem. Recombinant DNAse (rhDNAse) has been used with excellent results in CF. However, in non-CF bronchiectasis such results are not obtained. In a large multicentre trial by O'Donnell et al., rhDNAse was found to have detrimental effects in participants with worsening decline in lung function [33]. Forced vital capacity (FVC) was reduced by 3.1% compared to placebo. Patients also suffered an increase in the number of exacerbations in the intervention group. This finding is in contradistinction to the benefits documented in CF. This may have several explanations: firstly, there are differences in rheological properties of mucus in the CF airway when compared to the non-CF bronchiectatic airway [32]. Secondly, in CF, the pathology is mostly in the upper lobes, and the use of mucolytics may facilitate clearance with gravity, whilst in non-CF bronchiectasis the lower lobes are affected and this may hamper their effective clearance of thin secretions against gravity [33, 34]. Due to the harm demonstrated in this study, there have been no paediatric studies conducted in the use of rhDNAse. Therefore, the use of this drug is strongly discouraged in patients with non-CF bronchiectasis. The use of mucus hydrators like hypertonic saline and mannitol have been studied. Hypertonic saline has shown benefit in one small adult study when used in conjunction with chest physiotherapy [35]. A Cochrane review and a recent trial of the use of mannitol also have shown benefit in changing the physical properties of mucus in fourteen adults with bronchiectasis [36, 37]. 

Antibiotic therapy forms the cornerstone of bronchiectasis treatment. The use of antibiotics can prevent airway damage by treating infections, maintain and improve lung functions, and improve quality of life. Pseudomonas infection is rare in children with non-CF bronchiectasis [38]. Inhaled antibiotics have been extensively studied in the context of CF. The use of this strategy has the benefit of targeted drug delivery, limitation of systemic drug absorption, and reduction of side effects. The drug doses required for oral and intravenous antibiotics, to achieve bactericidal levels in airway secretions, need to be between 10 and 25 times above the mean inhibitory concentration. This, therefore, renders inhaled therapies a more attractive option in bronchiectasis. In order to have optimal use of inhaled drugs, they need to be at a pH above 4.0 and have an osmolarity between 100–1100 mOsmol. Several antibiotics, including tobramycin, ceftazidime, and gentamycin, have been studied especially in the context of CF in subjects colonized with *Pseudomonas aeruginosa *[39–41]. There is currently insufficient evidence for the recommendation of the use of inhaled antibiotics, especially since pseudomonas colonization is a rare event in non-CF bronchiectasis in children, although small studies with inhaled tobramycin, colistin, and aztreonam have suggested benefit [39].

Anti-inflammatory drugs like corticosteroids are a natural candidate in the management of bronchiectasis as they can play a pivotal role in breaking the cycle of inflammation. The anti-inflammatory effects are mediated by a reduction of inflammatory cytokines, inhibition of prostaglandins, reduction in adhesion molecules, and the inhibition of nitric oxide in the airway. Regrettably, systemic corticosteroids cannot be used long term due to their unfavourable side-effect profile. Inhaled corticosteroids have been shown in randomized trials to reduce the number of exacerbations, reduce sputum volume, and improve quality of life in bronchiectasis [22, 42, 43]. One randomized trial of eighty-six adults showed that subjects colonized with *Pseudomonas aeruginosa* derived the most benefit from the use of inhaled corticosteroids [22].

## 4. Macrolides and Bronchiectasis

Macrolide antibiotics are a group of antibiotics that contain a macrocytic lactone ring with a number of sugar moieties attached to these rings. Macrolides are further subclassified according to the number of lactone rings into the 14-, 15-, and 16-member ring macrolides (Table 1). The oldest of these drugs is erythromycin. Erythromycin is a 14-member macrolide, which was first isolated by McGiure and colleagues in 1952 from *Streptomyces erythreus* found in soil samples in the Philippines. The other macrolides are semisynthetic agents.

Azithromycin is an azalides with an added methyl-substituted nitrogen atom onto the lactone ring to form the 15-member ring. Clarithromycin is formed by the methylation of the hydroxyl group at position 6 of the lactone ring. These structural modifications confer azithromycin and clarithromycin a slightly better side effect profile when compared to erythromycin. These modifications reduce the interaction of these drugs with drugs metabolized by the cytochrome P450 system. There are also significantly fewer gastrointestinal side effects. Azithromycin and clarithromycin also have a far superior tissue penetration in vitro and a longer elimination half life and, thus, need once daily dosing. The drawback of the use of these agents is their significantly higher cost when compared to erythromycin, which is a relatively cheap and effective drug. Macrolide concentrations are at least 10-fold higher in epithelial lung fluid than in serum [44].

The mode of action of macrolides is by reversible binding to the 50 s subunit of the ribosome in prokaryocytes. This results in prevention of ribosomal translation and thus prevention of bacterial replication. Macrolides are bacteriostatic for *Staphylococci*, *Streptococci*, and *Haemophilus*, but they may exert bactericidal effects at very high concentrations. Macrolides do not have bactericidal effects against *Pseudomonas aeruginosa *but do result in inhibition of biofilm formation and also inhibit the organism's ability to produce toxins [45]. Macrolides are commonly used as a first-line therapy for treatment of acute bacterial infections such as community-acquired pneumonia in adults. The potential use of macrolides for their immune modifying effects was first discovered in patients with severe steroid dependent asthma [46]. The concomitant use of troleandomycin was found to result in significant improvement in asthma control in patients and also led to dose reduction of steroids without loss of asthma control. These immunomodulatory effects of macrolides are limited to the 14- and 15-membered ring macrolides.

The use of low-dose macrolides in the management of chronic inflammatory lung disease was initially found in Japanese patients with diffuse panbronchiolitis (DPB) [47–50]. DPB, a common condition in Japan and South East Asia, is a progressive inflammatory disorder whose sufferers present with chronic productive cough, wheezing, exertional dyspnoea, chronic sinusitis, mucoid *Pseudomonas aeruginosa* colonization, mixed restrictive and obstructive pulmonary functions, and diffuse chronic inflammation involving the bronchiolar and centrilobular regions of the airway. Untreated, DPB has a very poor prognosis; in 1984, the five-year survival rate was 26%. With the use of low dose erythromycin, the mortality of these patients was dramatically reduced with 10-year survival rates increasing to 92% [50]. This was coupled with an improvement in lung function and quality of life of sufferers. The immunomodulatory effects of macrolides are thought to result in reduction in sputum volume, inhibition of virulence factor production by bacteria, diminished neutrophil influx and downregulation of IL-8 production, inhibition of NF-*κβ* production, and reduction in both ICAM-1 and neutrophil elastase [51–54]. These immunomodulatory effects result in a reduction in pulmonary exacerbations, improved lung function, and improved quality of life [28, 55–61]. The clinical improvement of subjects may take up to three months to show an effect. 

The use of macrolides is not only limited to DPB. In the late 1990s, there was rekindled interest in the use of macrolides in the treatment of other chronic inflammatory lung disorder including CF. CF is a genetic disorder caused by a defect on chromosome 7, resulting in an abnormal CF transmembrane regulator gene, which results in an abnormal chloride secretion by the apical epithelial cells. The accumulation of aberrant CFTR in the endoplasmic reticulum is thought to result in calcium release and stimulation of NF*κβ*. NF*κβ* causes the release of IL-8 and inflammation of the airway. As the inflammatory process becomes chronic, there is histotoxic inflammation with an increase of lymphocytes and monocytes; this process occurs in the CF airway with continued predominance of neutrophils [62, 63]. It is thought that the chronic infections that occur in CF cause an increase in granulocyte colony stimulating factor (GCSF) and GM-CSF with signalling of reduction in cellular apoptosis causing this persistence of neutrophillic airway inflammation. In the setting of CF, azithromycin has been consistently found to result in a reduction in the number of pulmonary exacerbations, time to first exacerbation, and improvement in nutritional parameters [64–67]. In CF, macrolides form part of the cornerstone of therapy in subjects colonized with *Pseudomonas aeruginosa*, with emerging evidence of their benefit in CF subjects without *Pseudomonas aeruginosa *[68]. With initiation of macrolides, there is a modest initial improvement in lung functions.

There are a few studies looking at the immunomodulatory role of macrolides in the management of patients with non-CF bronchiectasis (Table 2). One adult study by Tsang et al. studied the effect of erythromycin in patients with severe idiopathic bronchiectasis. They found a significant improvement in FEV1, FVC, and sputum volume over a period of 8 weeks in 11 patients when compared to 10 controls [58]. In this study, there was no change in the proinflammatory mediators (IL-8, TNF-*α*, IL-1*αβ*, and leukotriene B4). Only one study in children showed an improvement on the small airways (maximal mid-expiratory flow) and a reduction in IL-8 [59]. The trials conducted on macrolides in bronchiectasis are limited in patient numbers and length of treatment but universally all have shown a consistent reduction in the frequency of exacerbations and sputum volumes [28, 57, 59, 60].

## 5. Macrolide Resistance and Safety

Long-term use of macrolides results in resistance particularly to *Streptococci*, *Haemophilus*, and *Staphylococci.* There are three mechanisms by which resistance occurs [69]. Firstly, this may be due to ribosomal target modification mediated by methylases encoded by the *erm*(B) gene. The second mechanism is due to mutation of the 23S rRNA or ribosomal proteins L4 and L22. This leads to conformational changes in the binding site of macrolides. Finally, active drug efflux occurs due to the membrane bound efflux protein *mef*(A) gene. Phaff et al. found increasing resistance of *S. aureus* to macrolides in CF patients, with an in resistance of 17.2% in those on macrolides versus 3.6% in CF subjects not on macrolides [70]. Tramper-Stranders et al. also found an exponential increase in *Staphylococcal* resistance to macrolides with increases from 83% in the first year of therapy to 100% in the third year of macrolide use [71].

There are safety concerns on the long-term use of macrolides. There is concern of cardiac side-effects (torsades de pointes) when using macrolides, particularly erythromycin, in conjunction with drugs that inhibit the CYP3A pathway. Postmarketing surveillance of the long-term use of erythromycin in Japan indicate this to be extremely rare [69]. The biggest concern with the use of macrolides is the development of resistant organisms, particularly the nontuberculous mycobacteria (NTM), which are commonly found in bronchiectasis patients. The newer macrolides azithromycin and clarithromycin form the backbone therapy for NTM management. It is known that carriage of NTM is high in bronchiectasis patients. A multicentre trial of CF subjects recovered NTM in 13% of over 900 subjects studied [72]. There is, therefore, a need for the development of novel macrolides that have no antimicrobial activity and only immunomodulatory properties.

## 6. Conclusion

Macrolides have immunomodulatory properties in addition to their anti-bacterial effects. The use of macrolides in non-CF-related bronchiectasis holds great promise as a therapeutic intervention that will not only affect the quality of life of sufferers but also act on the pathopysiological mechanism of bronchiectasis. More studies on the use of macrolides in this condition are needed to further ascertain their efficacy.

## Figures and Tables

**Table 1:** Types of macrolide antibiotics.

14-member ring macrolides	Erythromycin Troleandomycin Clarithromycin Roxithromycin

15-member ring macrolides	Azithromycin

16-member ring macrolides	Josamycin Spiramycin Midecamycin

**Table 2:** A summary of clinical trials of the use of macrolide therapy in bronchiectasis.

Author	Year	Study drug	Study design	Age group	Benefit
Tsang et al. [58]	1999	Erythromycin	RDBPCT	Adult	↑ FEV1, ↑ FVC ↓ sputum volume
Yalcin et al. [28]	2006	Clarithromycin	RPCT	Paediatric	↓ sputum volume, ↓ sputum cytokines
Koh et al. [59]	1997	Roxithromycin	RDBPCT	Adult	↓ airway reactivity to methacholine
Davies and Wilson [60]	2004	Azithromycin	Prospective open-label	Adult	↓ symptoms and ↑ D_LCO_
Cymbala et al. [57]	2005	Clarithromycin	Randomised open-label, crossover	Adult	↓ sputum volume
Serisier and Martin [55]	2011	Erythromycin	Retrospective RCT	Adult	↓ exacerbations ↓ antibiotic use
Coeman et al. [61]	2011	Erythromycin	Retrospective observational	Adult	Improved symptom score
Anwar et al. [56]	2008	Azithromycin	Retrospective observational	Adult	↑ FEV1 ↓ exacerbations

Abbreviations: ↑, increased, ↓, decreased; D_LCO_, pulmonary diffusion capacity for carbon monoxide; FVC, forced vital capacity; FEV1, forced expiratory volume in one second; RCT, randomised controlled trial; RDBCT, randomised double-blind controlled trial; RDBPCT, randomised double-blind placebo-controlled trial.

## References

[1] Laennac RTH, Forbes J (1962). A treatise on the disease of the chest. *Trans New York: Library of the New York Academy of Medicine*.

[2] Reid LM (1950). Reduction in bronchial subdivision in bronchiectasis. *Thorax*.

[3] Whitwell D (1952). A study of the pathology and pathogenesis of bronchiectasis. *Thorax*.

[4] Kothari NA, Kramer SS (2001). Bronchial diseases and lung aeration in children. *Journal of Thoracic Imaging*.

[5] Cole PJ (1986). Inflammation: a two-edged sword—the model of bronchiectasis. *European Journal of Respiratory Diseases*.

[6] Tsang KW, Chan KN, Ho PL (2000). Sputum elastase in steady-state bronchiectasis. *Chest*.

[7] Richman-Eisenstat JBY, Jorens PG, Hebert CA, Ueki I, Nadel JA (1993). Interleukin-8: an important chemoattractant in sputum of patients with chronic inflammatory airway diseases. *American Journal of Physiology*.

[8] Aldallal N, McNaughton EE, Manzel LJ (2002). Inflammatory response in airway epithelial cells isolated from patients with cystic fibrosis. *American Journal of Respiratory and Critical Care Medicine*.

[9] Rubin BK (2007). Mucus structure and properties in cystic fibrosis. *Paediatric Respiratory Reviews*.

[10] Zheng L, Lam WK, Tipoe GL (2002). Over expression of matrix metalloproteinases-8 and -9 in bronchiectasis airways in vivo. *European Respiratory Journal*.

[11] Säynäjäkangas O, Keistinen T, Tuuponen T, Klvelä S-L (1997). Bronchiectasis in Finland: trends in hospital treatment. *Respiratory Medicine*.

[12] Goeminne P, Dupont L (2010). Non-cystic fibrosis bronchiectasis: diagnosis and management in 21st century. *Postgraduate Medical Journal*.

[13] Field CE (1969). Bronchiectasis. Third report on a follow-up study of medical and surgical cases from childhood. *Archives of Disease in Childhood*.

[14] Singleton R, Morris A, Redding G (2000). Bronchiectasis in Alaska Native children: causes and clinical courses. *Pediatric Pulmonology*.

[15] Twiss J, Metcalfe R, Edwards E, Byrnes C (2005). New Zealand national incidence of bronchiectasis "too high" for a developed country. *Archives of Disease in Childhood*.

[16] Chang AB, Grimwood K, Mulholland EK, Torzillo PJ (2002). Bronchiectasis in Indigenous children in remote Australian communities. *Medical Journal of Australia*.

[17] Doğru D, Nik-Ain A, Kiper N (2005). Bronchiectasis: the consequence of late diagnosis in chronic respiratory symptoms. *Journal of Tropical Pediatrics*.

[18] Hayashi F, Means TK, Luster AD (2003). Toll-like receptors stimulate human neutrophil function. *Blood*.

[19] Akira S (2001). Toll-like receptors and innate immunity. *Advances in Immunology*.

[20] Simpson JL, Grissell TV, Douwes J, Scott RJ, Boyle MJ, Gibson PG (2007). Innate immune activation in neutrophilic asthma and bronchiectasis. *Thorax*.

[21] Mikami M, Llewellyn-Jones CG, Bayley D, Hill SL, Stockley RA (1998). The chemotactic activity of sputum from patients with bronchiectasis. *American Journal of Respiratory and Critical Care Medicine*.

[22] Tsang KW, Tan KC, Ho PL (2005). Inhaled fluticasone in bronchiectasis: a 12 month study. *Thorax*.

[23] Fuschillo S, De Felice A, Balzano G (2008). Mucosal inflammation in idiopathic bronchiectasis: cellular and molecular mechanisms. *European Respiratory Journal*.

[24] Chang AB, Redding GJ, Chernick V, Boat TF, Wilmott RW, Bush A (2006). Bronchiectasis. *Kendig’s Disorders of the Respiratory Tract in Children*.

[25] Parks WC, Wilson CL, López-Boado YS (2004). Matrix metalloproteinases as modulators of inflammation and innate immunity. *Nature Reviews Immunology*.

[26] Lanone S, Zheng T, Zhu Z (2002). Overlapping and enzyme-specific contributions of matrix metalloproteinases-9 and -12 in IL-13-induced inflammation and remodeling. *Journal of Clinical Investigation*.

[27] Taggart CC, Greene CM, Carroll TP, O’Neill SJ, McElvaney NG (2005). Elastolytic proteases: inflammation resolution and dysregulation in chronic infective lung disease. *American Journal of Respiratory and Critical Care Medicine*.

[28] Yalçin E, Kiper N, Özçelik U (2006). Effects of claritromycin on inflammatory parameters and clinical conditions in children with bronchiectasis. *Journal of Clinical Pharmacy and Therapeutics*.

[29] Feldman C (2011). Bronchiectasis: new approaches to diagnosis and management. *Clinics in Chest Medicine*.

[30] Elkins MR, Jones A, van der Schans C (2006). Positive expiratory pressure physiotherapy for airway clearance in people with cystic fibrosis. *Cochrane Database of Systematic Reviews*.

[31] Murray MP, Pentland JL, Hill AT (2009). A randomised crossover trial of chest physiotherapy in non-cystic fibrosis bronchiectasis. *European Respiratory Journal*.

[32] Bush A, Payne D, Pike S, Jenkins G, Henke MO, Rubin BK (2006). Mucus properties in children with primary ciliary dyskinesia: comparison with cystic fibrosis. *Chest*.

[33] O'Donnell AE, Barker AF, Ilowite JS, Fick RB (1998). Treatment of idiopathic bronchiectasis with aerosolized recombinant human DNase I. *Chest*.

[34] Karadag B, Karakoc F, Ersu R, Kut A, Bakac S, Dagli E (2005). Non-cystic-fibrosis bronchiectasis in children: a persisting problem in developing countries. *Respiration*.

[35] Kellett F, Redfern J, McL Niven R (2005). Evaluation of nebulised hypertonic saline (7%) as an adjunct to physiotherapy in patients with stable bronchiectasis. *Respiratory Medicine*.

[36] Daviskas E, Anderson SD, Young IH (2010). Effect of mannitol and repetitive coughing on the sputum properties in bronchiectasis. *Respiratory Medicine*.

[37] Wills P, Greenstone M (2006). Inhaled hyperosmolar agents for bronchiectasis. *Cochrane Database of Systematic Reviews*.

[38] Kapur N, Masters IB, Chang AB (2009). Exacerbations in noncystic fibrosis bronchiectasis: clinical features and investigations. *Respiratory Medicine*.

[39] Rubin BK (2008). Aerosolized antibiotics for non-cystic fibrosis bronchiectasis. *Journal of Aerosol Medicine and Pulmonary Drug Delivery*.

[40] Orriols R, Roig J, Ferrer J (1999). Inhaled antibiotic therapy in non-cystic fibrosis patients with bronchiectasis and chronic bronchial infection by *Pseudomonas aeruginosa*. *Respiratory Medicine*.

[41] Scheinberg P, Shore E (2005). A pilot study of the safety and efficacy of tobramycin solution for inhalation in patients with severe bronchiectasis. *Chest*.

[42] Martínez-García MA, Perpiñá-Tordera M, Román-Sánchez P, Soler-Cataluña JJ (2006). Inhaled steroids improve quality of life in patients with steady-state bronchiectasis. *Respiratory Medicine*.

[43] Kapur N, Bell S, Kolbe J, Chang AB (2009). Inhaled steroids for bronchiectasis. *Cochrane Database of Systematic Reviews*.

[44] Togami K, Chono S, Morimoto K (2011). Distribution characteristics of clarithromycin and azithromycin, macrolide antimicrobial agents used for treatment of respiratory infections, in lung epithelial lining fluid and alveolar macrophages. *Biopharmaceutics & Drug Disposition*.

[45] Shinkai M, Henke MO, Rubin BK (2008). Macrolide antibiotics as immunomodulatory medications: proposed mechanisms of action. *Pharmacology and Therapeutics*.

[46] Spector SL, Katz FH, Farr RS (1974). Troleandomycin: effectiveness in steroid dependent asthma and bronchitis. *Journal of Allergy and Clinical Immunology*.

[47] Nagai H, Shishido H, Yoneda R, Yamaguchi E, Tamura A, Kurashima A (1991). Long-term low-dose administration of erythromycin to patients with diffuse panbronchiolitis. *Respiration*.

[48] Tredaniel J, Zalcman G, Gerber F (1993). Diffuse panbronchiolitis: efficacy of low-dose erythromycin. *Respiratory Medicine*.

[49] Hoiby N (1994). Diffuse panbronchiolitis and cystic fibrosis: east meets West. *Thorax*.

[50] Kudoh S, Azuma A, Yamamoto M, Izumi T, Ando M (1998). Improvement of survival in patients with diffuse panbronchiolitis treated with low-dose erythromycin. *American Journal of Respiratory and Critical Care Medicine*.

[51] Takizawa H, Desaki M, Ohtoshi T (1997). Erythromycin modulates IL-8 expression in normal and inflamed human bronchial epithelial cells. *American Journal of Respiratory and Critical Care Medicine*.

[52] Khair OA, Devalia JL, Abdelaziz MM, Sapsford RJ, Davies RJ (1995). Effect of erythromycin on *Haemophilus influenzae* endotoxin-induced release of IL-6, IL-8 and sICAM-1 by cultured human bronchial epithelial cells. *European Respiratory Journal*.

[53] Gorrini M, Lupi A, Viglio S (2001). Inhibition of human neutrophil elastase by erythromycin and flurythromycin, two macrolide antibiotics. *American Journal of Respiratory Cell and Molecular Biology*.

[54] Taggart C, Coakley RJ, Greally P, Canny G, O’Neill SJ, McElvaney NG (2000). Increased elastase release by CF neutrophils is mediated by tumor necrosis factor-*α* and interleukin-8. *American Journal of Physiology*.

[55] Serisier DJ, Martin ML (2011). Long-term, low-dose erythromycin in bronchiectasis subjects with frequent infective exacerbations. *Respiratory Medicine*.

[56] Anwar GA, Bourke SC, Afolabi G, Middleton P, Ward C, Rutherford RM (2008). Effects of long-term low-dose azithromycin in patients with non-CF bronchiectasis. *Respiratory Medicine*.

[57] Cymbala AA, Edmonds LC, Bauer MA (2005). The disease-modifying effects of twice-weekly oral azithromycin in patients with bronchiectasis. *Treatments in Respiratory Medicine*.

[58] Tsang KWT, Ho PI, Chan KN (1999). A pilot study of low-dose erythromycin in bronchiectasis. *European Respiratory Journal*.

[59] Koh YY, Lee MH, Sun YH, Sung KW, Chae JH (1997). Effect of roxithromycin on airway responsiveness in children with bronchiectasis: a double-blind, placebo-controlled study. *European Respiratory Journal*.

[60] Davies G, Wilson R (2004). Prophylactic antibiotic treatment of bronchiectasis with azithromycin. *Thorax*.

[61] Coeman M, Van Durme Y, Bauters F (2011). Neomacrolides in the treatment of patients with severe asthma and/or bronchiectasis: a retrospective observational study. *Therapeutic Advances in Respiratory Disease*.

[62] Eller J, Lapa e Silva JR, Poulter LW, Lode H, Cole PJ (1994). Cells and cytokines in chronic bronchial infection. *Annals of the New York Academy of Sciences*.

[63] Loukides S, Bouros D, Papatheodorou G, Lachanis S, Panagou P, Siafakas NM (2002). Exhaled H_2_O_2_ in steady-state bronchiectasis: relationship with cellular composition in induced sputum, spirometry, and extent and severity of disease. *Chest*.

[64] McCormack J (2002). Effect of long term treatment with azithromycin on disease parameters in cystic fibrosis: a randomised trial. *Thorax*.

[65] Equi A, Balfour-Lynn IM, Bush A, Rosenthal M (2002). Long term azithromycin in children with cystic fibrosis: a randomised, placebo-controlled crossover trial. *Lancet*.

[66] Saiman L, Marshall BC, Mayer-Hamblett N (2003). Azithromycin in patients with cystic fibrosis chronically infected with *Pseudomonas aeruginosa*. A randomized controlled trial. *Journal of the American Medical Association*.

[67] Clement A, Tamalet A, Leroux E, Ravilly S, Fauroux B, Jais JP (2006). Long term effects of azithromycin in patients with cystic fibrosis: a double blind, placebo controlled trial. *Thorax*.

[68] Saiman L, Anstead M, Mayer-Hamblett N (2010). Effect of azithromycin on pulmonary function in patients with cystic fibrosis uninfected with *Pseudomonas aeruginosa*: a randomized controlled trial. *Journal of the American Medical Association*.

[69] Kanoh S, Rubin BK (2010). Mechanisms of action and clinical application of macrolides as immunomodulatory medications. *Clinical Microbiology Reviews*.

[70] Phaff SJ, Tiddens HAWM, Verbrugh HA, Ott A (2006). Macrolide resistance of Staphylococcus aureus and Haemophilus species associated with long-term azithromycin use in cystic fibrosis. *Journal of Antimicrobial Chemotherapy*.

[71] Tramper-Stranders GA, Wolfs TFW, Fleer A, Kimpen JLL, Van Der Ent CK (2007). Maintenance azithromycin treatment in pediatric patients with cystic fibrosis: long-term outcomes related to macrolide resistance and pulmonary function. *Pediatric Infectious Disease Journal*.

[72] Olivier KN, Weber DJ, Wallace RJ (2003). Nontuberculous mycobacteria—I: multicenter prevalence study in cystic fibrosis. *American Journal of Respiratory and Critical Care Medicine*.

